# Overall survival in metastatic melanoma correlates with pembrolizumab exposure and T cell exhaustion markers

**DOI:** 10.1002/prp2.808

**Published:** 2021-06-15

**Authors:** Vishal Navani, Moira C. Graves, Giovana Celli Marchett, Hiren Mandaliya, Nikola A. Bowden, Andre van der Westhuizen

**Affiliations:** ^1^ Calvary Mater Hospital Newcastle Waratah NSW Australia; ^2^ Centre for Drug Repurposing and Medicines Research University of Newcastle and Hunter Medical Research Institute Callaghan NSW Australia

**Keywords:** CXCR6, drug exposure, immune checkpoint blockade, metastatic melanoma, pembrolizumab, pharmacokinetics, TIM‐3

## Abstract

Trial data support an absence of an exposure–survival relationship for pembrolizumab. As these relationships remain unexamined in a real‐world setting, we determined them in metastatic melanoma prospectively in an observational study. Translational objectives included identifying biomarkers of progressive disease (PD). Checkpoint blockade naïve patients receiving 2 mg/kg Q3W pembrolizumab had pharmacokinetic and clinical outcome data collected. Trough, a valid surrogate for drug exposure, was assessed using ELISA. T‐cell exhaustion and chemokine markers were determined using flow cytometry. Geometric means of exposures and biomarkers were tested against objective response groups using one‐way ANOVA. The cohort was split by the median into high versus low pembrolizumab exposure groups. Kaplan–Meier progression‐free survival (PFS) and overall survival (OS) curves were estimated for high versus low exposure, compared using the log rank test. The high pembrolizumab exposure group (*n* = 14) experienced substantially longer median OS (not reached vs. 48 months, *p* = .014), than the low exposure group (*n* = 14). A similar positive exposure PFS relationship was found (median not reached vs. 48 months, *p* = .045). The frequency of TIM‐3 expression on CD4^+^ T cells was significantly higher in PD (mean 27.8%) than complete response (CR) (13.38%, *p* = .01) and partial response (12.4%, *p* = .05). There was a near doubling of CXCR6 and TIM‐3 co‐expression on CD4^+^ T cells in PD (mean 23.3%) versus CR (mean 11.4, *p* = .003) and partial response (9.8%, *p* = .0001). We describe positive exposure‐PFS and exposure‐OS relationships for pembrolizumab in metastatic melanoma. TIM‐3, alongside co‐expression of CXCR6 and TIM‐3 on circulating CD4^+^ T cells are potential bio markers of treatment failure.

## Graphical abstract

Low pembrolizumab exposure is associated with inferior overall survival in metastatic melanoma.

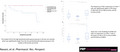

**Abbreviations**
BOR: best overall response
CR: complete response
ICB: immune checkpoint blockade
iRECIST: immune response evaluation criteria in solid tumors
mAb: monoclonal antibody
NSCLC: non‐small cell lung cancer
OS: overall survival
PBMC: peripheral blood mononuclear cell
PD: progressive disease
PD‐1: programmed cell death 1
PD‐L1: programmed death ligand 1
PD‐L2: programmed death ligand 2
PFS: progression‐free survival
PK: pharmacokinetic
PR: partial response
SD: stable disease

## 1 INTRODUCTION

Targeting the inhibitory interaction between T cell checkpoint, programmed cell death 1 (PD‐1), and its tumoral and stromal ligands, PD‐L1/PD‐L2 has transformed outcomes across oncological indications.^1^ The immunosuppressive tumor microenvironment potentiates T cell exhaustion and restrains the anti‐tumoral immune response.^2^ Restoration of deficient anti‐tumoral immunity by pembrolizumab, a humanized anti‐PD‐1 IgG4 monoclonal antibody (mAb), is an established standard of care that has led to durable responses in metastatic melanoma.^3^ However, there is a need to understand the factors driving failure of immune checkpoint blockade (ICB) in order to help the majority of patients that do not respond to these agents.^4^

Pembrolizumab has pharmacokinetic (PK) similarities with other large molecular weight ICB mAbs; a low central volume of distribution, linear PKs at clinically relevant doses, confinement primarily to the vascular compartment, and a prolonged half‐life.^5^ The seamless trial design of multiple expansion cohorts when promising early efficacy was first noted,^6^ together with lack of dose‐limiting toxicities, meant that the traditional approach of obtaining a maximum tolerated dose to guide pivotal registrational trials was not undertaken.^7^ Subsequently, in silico PK and pharmacodynamic (PD) studies were key for regimen selection.^8^

Modeling pharmacodynamic data found that peripheral target saturation for pembrolizumab begins at 1 mg/kg Q3W with a steady‐state dose of 2 mg/kg Q3W reaching a 90% probability of 95% target engagement,^9^ suggesting a flat dose–response relationship in the clinic. Maximal lymphocyte stimulation was seen around 1 mg/kg.^10^ Trial data supported an absence of a dose or exposure–response relationship at clinically relevant doses^11^ and suggested that the classic clinicopathologic features known to influence mAB did not affect pembrolizumab PKs in a clinically meaningful manner. This inferred limitations to inter‐patient variability.^12^ However, prospective real‐world data with another anti‐PD1 mAb, nivolumab, in metastatic non‐small cell lung cancer (NSCLC) and melanoma found gender, baseline albumin, and body surface area affected PKs to a clinically meaningful extent, throwing previous assumptions into doubt.^13^

Real‐world data regarding exposure–response relationships with ICB conflict with the trial evidence. A cohort of pre‐treated metastatic NSCLC patients given 3 mg/kg Q2W nivolumab found patients with higher exposures of drug defined by trough measurements had notably improved best overall response (BOR) *p* = .002 and overall survival (OS) *p* = .001.^14^ Trough concentrations are a regulatory body approved surrogate for drug exposure.^15^

There are no clinically validated biomarkers to identify early resistance or predict lack of response to pembrolizumab in metastatic melanoma. TIM‐3 is another immune checkpoint that marks the most terminally exhausted subset of CD8^+^ tumor infiltrating lymphocytes.^16^ The exact mechanism by which TIM‐3 contributes to T cell dysfunction remains to be defined, but may involve antagonism of the T cell stem‐like state and decreased CD8^+^ differentiation.^17^ TIM‐3 expression has been associated with rapid tumor progression, and our previous work highlighted that high TIM‐3 expression on CD8^+^ T cells was associated with poor treatment response to ICB.^18^

Chemokines are chemotactic cytokines that regulate leukocyte trafficking. Chemokine CXCL16 and its T cell ligand CXCR6 are also key propagators of melanoma.^19^ CXCL16 can be expressed by malignant cells as a transmembrane molecule and mediate effect via autocrine binding to CXCR6.^20^ The CXCR6/CXCL16 axis is pro‐inflammatory,^21^ CXCR6 is expressed by a self‐renewing subset of melanoma stem cells,^22^ and melanoma secretes CXCL16, contributing to CXCR6‐mediated leukocyte recruitment.^19^ We previously identified patients with disease progression on pembrolizumab had consistently higher proportions of CD4^+^ and CD8^+^ T cells‐expressing CXCR6.^18^

Given the number of confounders at play, both patient (inter‐patient variability in plasma exposures and clearance) and malignancy (histopathology, tumor burden, immunogenicity) related, it is challenging to unravel whether lower plasma exposures of ICB are cause or effect of a lack of response. PK and pharmacodynamic relationships have been primarily studied in the peripheral circulation which has questionable relevance to the tumor microenvironment.^23^

We aimed to determine the relationship between pembrolizumab drug exposure and clinical outcomes such as BOR, progression‐free survival (PFS), and OS in patients with metastatic melanoma in a real‐world setting. We also sought to identify whether circulating T cell exhaustion markers and specific chemokines may help to identify patients with progressive disease (PD).

## 2 METHODS

### 2.1 Participants and treatment

This study was approved by local institutional review boards. Patients gave informed written consent. Individuals with metastatic melanoma receiving 2 mg/kg Q3W pembrolizumab had serial PK trough blood draws ≤48 h prior to their next scheduled dose, up to a maximum of 22 cycles. Peripheral blood mononuclear cells (PBMCs) and plasma were harvested from the same draws and stored at −80°C.

All patients were treated with pembrolizumab administered over 30 min intravenously. Treatment was continued until the physician assessed disease progression (clinically or radiologically), patient decision to cease treatment or unacceptable toxicity. Treatment discontinuation in the context of sustained complete response (CR) was allowed at the discretion of the treating physician.

### 2.2 Study endpoints

Pharmacokinetic data, PBMCs, patient baseline characteristics, clinical outcome data and BOR, PFS, and OS were collected prospectively. OS was defined as time from first pembrolizumab initiation until death from any cause. PFS was defined as the time from pembrolizumab initiation until documented progression (clinical or radiological) or death from any cause.

Imaging assessment was undertaken according to immune response evaluation criteria in solid tumors (iRECIST)^24^ by 2 unblinded investigators (VN and AvW) and confirmed by a separate blinded investigator (HM). BOR groups were defined as CR, partial response (PR), stable disease (SD), and progressive disease (PD). Contrast‐enhanced computerized tomography scanning was used for the imaging assessments.

### 2.3 Plasma concentrations

Plasma trough pembrolizumab concentrations were determined using the Abcam^®^ pembrolizumab ELISA kit as per manufacturer instructions. The lower limit of detection was 10 ng/ml.^25^ The mean of duplicate biological plasma samples was used for each timepoint. Geometric mean trough concentrations were calculated for this continuous variable.

### 2.4 T‐cell marker immune subsets

The immune subsets based on T‐cell markers were determined using Flow Cytometry on the BD Science (Becton, Dickinson and Company) Fortessa ×20 as previously described.^18^ Frozen PBMCs were thawed and stained with the following targets: horizon Fixable Viability stain 575V, Hu CD3 BUV737 UCHT1, Hu CD4 BUV496 SK3, Hu CD8 APC‐H7 SK1, Hu CD279(PD‐1) BB515 EH12.1, Hu TIM‐3(CD366) Alexa 647 7D3, Hu LAG‐3(CD223) APC‐R700T47‐530. Samples were gated to acquire 50,000 live cell events.

The samples were gated for lymphocytes, single cells, and then live cells. The CD3 subset was gated as the fluorophore (BUV737) versus side scatter (SSE). To separate into CD4^+^ and CD8^+^ subsets, all positive CD3^+^ cells were then further divided into CD4^+^ and CD8^+^ by gating CD3 versus CD4^+^ (BUV496) or CD8^+^ (APC‐H7 SK1).

For each of the other surface markers (e.g., TIM3), positive CD4 or CD8 were gated against the other surface markers e.g., CD4^+^ (BUV496 SK3) versus TIM‐3 (Alexa 647 7D3).

### 2.5 Statistical analysis

Descriptive statistics included mean, range, and standard deviation of the continuous baseline patient characteristics. Non‐parametric correlation analysis between clinical characteristics was performed using Spearman's rho.

Statistically significant differences in clinical characteristics, plasma pembrolizumab concentrations, and T‐cell markers between the BOR groups were tested for using one‐way ANOVA with Bonferroni correction.

The cohort was split into high versus low pembrolizumab exposure groups, divided by the median trough. Kaplan–Meier survival analysis for PFS and OS was undertaken with the logrank test used to compare survival between pembrolizumab exposure groups. Due to the signal seeking nature of this early observational work, no formal pre‐specified statistical power calculations were undertaken to compare results between exposure groups. Statistical analysis was performed using IBM SPSS Statistics V27. A two‐sided *p*‐value < .05 was considered statistically significant.

### 2.6 Nomenclature of targets and ligands

Key protein targets and ligands in this article are hyperlinked to the corresponding entries in http://www.guidetopharmacology.org, the common portal for data from the IUPHAR/BPS guide to pharmacology,^26^ and are permanently archived in the concise guide to pharmacology 2019/20.^27^

## 3 RESULTS

Clinical characteristics and demographics are summarized in Table 1 (range) [standard deviation] 28 patients participated, 5 patients ceased treatment due to PD or death, 4 patients were BRAF V600E positive, all pre‐treated with BRAF inhibitor/MEK inhibitor combinations. Median follow up was 32.5 months (range = 2–54 months). PFS and OS were statistically significantly correlated (*r* = .885, *p* = .0001) and both PFS and OS negatively correlated with baseline lactate dehydrogenase (LDH) prognostically (*r* = −.620, *p* = .001 and *r* = −.516, *p* = .006, respectively). There was no statistically significant correlation between other known prognostic variables such as age at commencement of pembrolizumab, eastern co‐operative group (ECOG) or LDH, and the measured pembrolizumab trough concentrations. Age at diagnosis of primary melanoma, age of diagnosis of metastatic disease and baseline LDH were not different between the high and low pembrolizumab exposure groups (Table 1).

**TABLE 1:** Patient characteristics by pembrolizumab exposure group

	High pembrolizumab exposure *N* = 14	Low pembrolizumab exposure *N* = 14	*N*	*p* value two‐tailed
Age of primary diagnosis	63 (40–75)	64.5 (32–82)	11	.765
			14	
Age of metastatic disease	65.4 (45–78)	69.93 (46–82)	14	.24
			14	
LDH baseline (U/L)	200.38 [58.7]	214.93 [92.5]	14	.633
BRAF V600E+ve	1	3	4	
ECOG 0	6	8	14	
ECOG 1	6	7	13	
M1a	2	3	5	
M1b	6	5	11	
M1c	3	2	5	
M1d	3	4	7	
CR	7	4	11	
PR	3	7	10	
SD	2	0	2	
PD	2	3	5	

Abbreviations: CR, complete response; PD, progressive disease; PR, partial response; SD, stable disease.

### 3.1 Plasma trough concentrations and BOR

**FIGURE 1:**
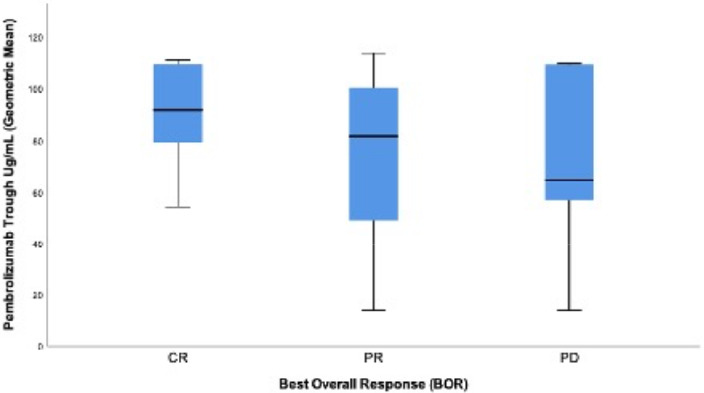
Pembrolizumab plasma trough concentrations (geometric mean) grouped by best overall response. CR, PR and PD were determined by immune response evaluation criteria in solid tumors. CR patients had higher pembrolizumab trough concentrations than PR patients and PD patients which did not reach significance. CR, complete response; PD, progressive disease; PR, partial response

The geometric mean pembrolizumab plasma trough concentrations across all timepoints were not a statistically significant difference between the BOR groups. The number of participants with SD (*n* = 2) was too small for statistical analysis. Trends observed included CR (*n* = 11) with 34.5% higher geometric mean pembrolizumab trough concentrations (90.8 mcg/ml) than PR (*n* = 10) (67.5 mcg/ml, *p* = ns). CR had 27.8% higher trough concentrations than PD (*n* = 5) (71.5 mcg/ml, *p* = ns). SD (*n* = 2) had mean trough pembrolizumab concentrations of 106.4 mcg/ml. The median pembrolizumab plasma concentrations for each BOR followed the same trend of being higher in the CR (91.8 mcg/ml) and PR (81.4 mcg/ml) groups compared with PD (64.7 mcg/ml), with no statistically significant difference, but the trough concentration variability in exposures was high within the groups (Figure 1).

### 3.2 Plasma trough concentrations and OS/PFS

**FIGURE 2:**
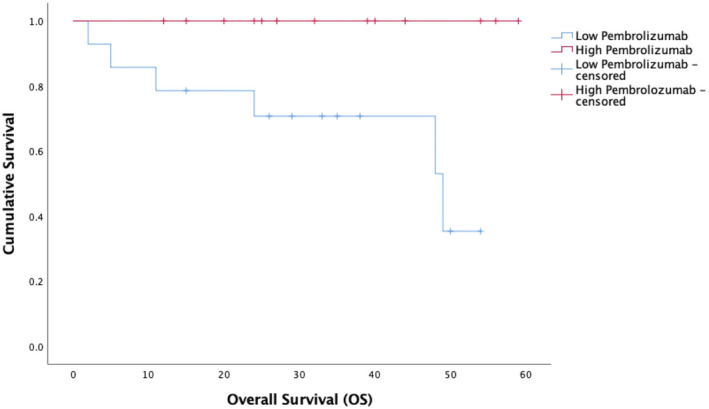
Overall survival (OS) Kaplan–Meier survival curves for high pembrolizumab exposure (red) compared to low pembrolizumab exposure concentrations (blue) groups. The median OS for high pembrolizumab exposure group was not reached, which was significantly longer than the low pembrolizumab exposure median of 48 months (*p* = .014)

**FIGURE 3:**
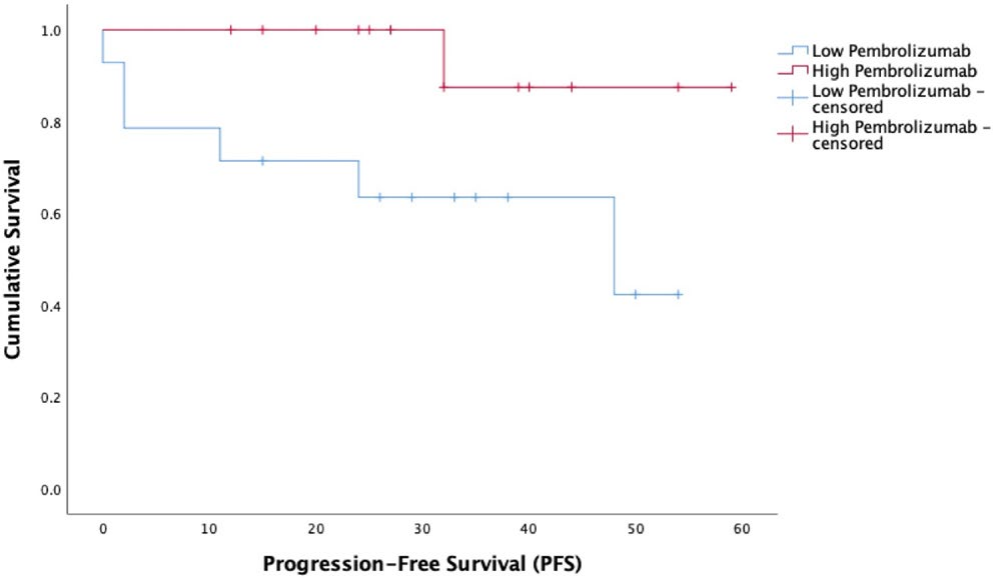
Progression‐free survival (PFS) Kaplan–Meier survival curves for high pembrolizumab exposure (red) compared to low pembrolizumab exposure (blue) groups. The median PFS for the high pembrolizumab exposure group was not reached, which was significantly longer than low pembrolizumab exposure median PFS of 48 months (*p* = .045)

The high pembrolizumab exposure group (geometric mean trough concentration = 55.9 ± 25.6 mcg/ml, *n* = 14) experienced meaningfully longer OS than the low exposure group (geometric mean trough concentration = 104.2 ± 8.1 mcg/ml, *n* = 14) with median OS not reached versus 48 months (*p* = .014) (Figure 2). A similar positive exposure PFS relationship was found (median not reached vs. 48 months, *p* = .045) (Figure 3).

### 3.3 Pembrolizumab trough concentrations, T‐cell exhaustion, and chemokine markers

There were no statistically significant or clinically meaningful associations between pembrolizumab exposure groups and upregulation of T‐cell exhaustion or chemokine markers over time.

### 3.4 T‐cell exhaustion markers, chemokines, and BOR

#### 3.4.1 TIM‐3

**FIGURE 4:**
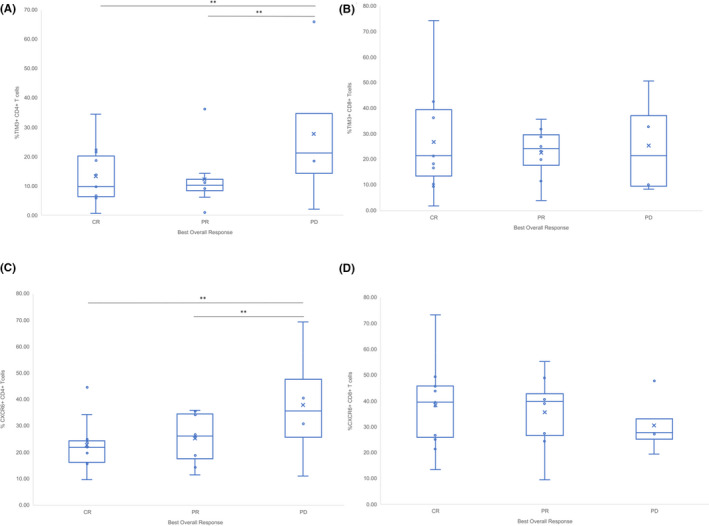
Mean frequency of TIM‐3 and CXCR6 on the surface of CD4^+^ and CD8^+^ T cells in best overall response groups. (A) Percentage of TIM‐3^+^CD4^+^ T cells; (B) Percentage of TIM‐3^+^CD8^+^ T cells; (C) Percentage of CXCR6^+^CD4^+^ T cells; (D) Percentage of CXCR6^+^CD8^+^ T cells in Best Overall Response groups. Complete response (CR), progressive disease (PD), partial response (PR). *p < .05, **p < .01, x = mean

The frequency of TIM‐3 expression on CD4^+^ T cells was increased in absolute terms by over 10% in PD (mean 27.75%, CI 6.26%–54.19%) than CR (13.38%, CI 8.26%–19.18%) (*p* = .01) and PR (mean 12.36%, CI 6.70%–20.24%) (*p* = .05) (Figure 4A). TIM‐3 on the surface of CD8^+^ T cells was similar in PD (mean 25.42%, CI 8.68%–41.72%) than PR (mean 22.55%, CI 15.93%–28.90%) and CR (26.87%, CI 15.87%–28.90%) (*p* = .14) (Figure 4B).

#### 3.4.2 CXCR6

There was a higher frequency of CXCR6 expression on CD4^+^ T cells for PD (mean 37.93%, CI 15.96%–59.77%) compared with CR (mean 22.76%, CI 17.72%–28.89%) (*p* = .002) and PR (25.34%, CI 18.63%–31.03%) (*p* = .001) (Figure 4C). There was no significant difference between expression of CXCR6 on the surface of CD8^+^ T cells in CR compared with PR or PD (Figure 4D).

### 3.5 Co‐expression of CXCR6 and T‐cell exhaustion markers

**FIGURE 5:**
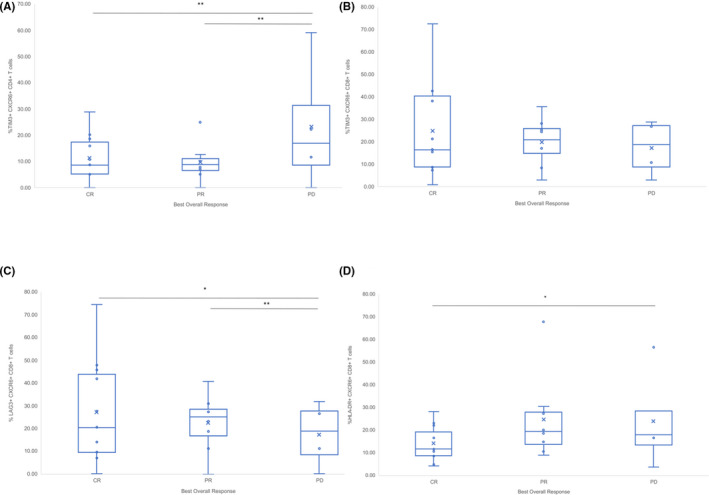
Frequency of CXCR6 and TIM‐3, LAG3 and HLA‐DR on the surface of CD4^+^ and CD8^+^ T cells in best overall response (BOR) groups. (A) Frequency of CXCR6 and TIM‐3 on CD4^+^ T cells, (B) Frequency of CXCR6 and TIM‐3 on CD8^+^ T cells, (C) Frequency of CXCR6 and LAG3 on CD8^+^ T cells, (D) Frequency of CXCR6 and HLA‐DR on CD8^+^ T cells. Complete response (CR), progressive disease (PD), partial response (PR). *p < .05, **p < .01, x = mean

There was increased frequency of co‐expression of CXCR6 and TIM3 on the surface of CD4^+^ T cells for PD (mean 23.29%, CI 5.6–47.28%–26.06%) compared with CR (mean 11.37%, CI 6.66%–16.70%) (*p* = .003) and PR (mean 9.83%, CI 5.91%–14.40%) (*p* = .0001) (Figure 5A). This co‐expression relationship was not seen on the surface of CD8^+^ T cells for PD (mean 17.32%, CI 6.82%–27.82%) compared with PR (mean 19.95% CI 13.34%–26.52%) or CR (mean 11.37%, CI 6.66%–16.70%) (*p* = ns) (Figure 5B).

We found downregulated frequency of co‐expression of CXCR6 and LAG3 on the surface of CD8^+^ T cells in the PD cohort (mean 17.38%, CI 5.61%–29.14%) when compared with the PR cohort (mean 22.44%, CI 14.31%–30.17%) (*p* = .021) and CR (mean 27.15%, CI 14.76%–40.44%) (*p* = .005) (Figure 5C). The PD cohort was enriched for a higher frequency of co‐expression of CXCR6 and HLA‐DR on the surface of CD8^+^ T cells (mean 24.04%, CI 7.57%–46.64%) versus CR (mean 11.37%, CI 6.83%–16.69%) (*p* = .001) (Figure 5D). There was no statistically significant difference between BOR groups for co‐expression of CXCR6 and PD‐L1 on CD4^+^ or CD8^+^ T cells.

## 4 DISCUSSION

Evasion of immune surveillance is an emerging hallmark of cancer.^28^ Metastatic melanoma is the archetypal immunosensitive malignancy. The high tumoral mutational burden induced by ultraviolet light, brisk stimulation of an innate response by malignant melanocytes, and dense lymphocytic infiltration throughout the tumor microenvironment are well established.^29^

Pembrolizumab can maintain durable responses in advanced melanoma,^30^ with 5 year survival rates approaching 40%. However predictive biomarkers of response remain elusive, which is key to intensifying or altering treatment lines for the majority of patients that still do not benefit from ICB. In this context, identification of such factors that contribute to the stark inter‐patient variability in clinical outcomes is critical. We identified a clinically and statistically significant drug exposure and OS signal for pembrolizumab in a real‐world setting. Similar results were seen with high drug exposure and PFS. Given the known association of these clinical endpoints with several prognostic factors such as ECOG and baseline LDH,^31^ the results are suggestive of a qualitative exposure–survival relationship only, given the univariate analysis was not corrected for potential imbalances in baseline prognostic factors between the exposure groups due to the influence of sample size.

A real‐world identification of a clinically meaningful relationship between drug exposure and response for nivolumab in second‐line metastatic NSCLC has been described.^14^ Partial responders, 10 weeks post commencement of nivolumab, had 73% higher (*p* = .002) trough concentrations than non‐responders. Patients with higher trough concentrations also experienced meaningfully longer median OS (not reached vs. 306 days, *p* = .001),^14^ although this also did not undergo correction for prognostic variables due to the small sample size. No relationship was found between higher drug exposure and PFS. Due to the lack of association between immune‐related adverse events and drug exposure, the authors suggested dose intensification for patients with low trough concentrations early in their disease course.

Our prospective work, with serial sampling of draws, consistent weight‐based dosing, representative real‐world cohort of patients, and long duration of follow‐up offers confidence in data relevance and applicability. Though peripheral PD‐1 receptor saturation is achieved at clinically utilized dose levels of pembrolizumab,^9^ the relationship between peripheral and intra‐tumoral PD‐1 receptor occupancy, and subsequent restoration of anti‐tumor T cell immunity has not been established across ICB.^32^ Therefore peripheral blood mononuclear cell receptor occupancy may be of limited clinical relevance when examining the exposure–efficacy relationship. Sponsor data with nivolumab in the metastatic melanoma setting noted that patients with an objective imaging response had higher nivolumab exposures than non‐responders within dose levels, suggesting drug exposure may predict for efficacy.^32^ Mechanistically, anti‐PD‐1 targeting ICBs are thought to be restricted to restoration of anti‐tumoral immunity for effector T cells already present within the tumor microenvironment. There is minimal impact on promoting an increase in intra‐tumoral lymphocyte trafficking in melanoma.^33^ Therefore, a subsequent requirement for IgG mAbs such as pembrolizumab to overcome disordered tumor vasculature in order to re‐invigorate exhausted T effector cells within the tumor microenvironment may require higher drug exposure than necessary to saturate the T cell PD‐1 receptor in the peripheral circulation. Ipilimumab, the first approved ICB, has clear dose and exposure‐dependent PKs associated with clinical outcomes. It acts via a separate mechanistic inhibition of cytotoxic T‐lymphocyte‐associated protein 4, leading to expansion of cognate CD8^+^ T cells in tumor draining lymph nodes.^34^ Clinical efficacy increased with plasma exposure, again defined by higher trough concentrations. Pooled data from four phase II studies revealed patients at the 95th percentile of trough (103 mcg/ml) had an OS HR of .55 relative to patients with a trough at the median.^35^, ^36^ A dose–survival relationship noted that pooled analysis was confirmed in a randomized phase III study, with an improvement in mOS from a 10 mg/kg versus 3 mg/kg Q3W dosing (15.7 vs. 11.5 months, HR .84, *p* = .04), albeit at a cost of higher adverse events.^35^

Our results are discordant with available pembrolizumab trial data. Pembrolizumab was evaluated across 2 mg/kg Q3W–10 mg/kg Q2W/3W schedules, with a 2 mg/kg Q3W efficacy plateau identified in metastatic NSCLC. Characterizations of variations in pembrolizumab exposures, defined by area under the curve steady‐state at 6 weeks, across these regimens had no statistically significant effect on tumor response. About 2 mg/kg Q3W was deemed to provide sufficient safe anti‐tumor activity, suggesting a wide therapeutic index.^37^ This regimen was advanced across indications including metastatic melanoma. Given the enriched patient populations seen in clinical trials, it is unsurprising that the estimates for residual error for plasma exposures of ICB in the real‐world ranges from 16% to 27%,^5^ and this emphasizes the importance of real‐world studies that account for inter‐individual variations in drug exposure to frame the exposure efficacy discussion.

Our work and that of Basak^14^ cannot uncouple clearance from exposure, given the solitary dose level and thus assumption of perfect correlation between clearance and exposure. The clearance of pembrolizumab varies over time, with lower clearance at steady‐state and faster clearance of drug in non‐responders.^38^ Turner reported a flat dose–exposure response curve, with no relationship across a fivefold dose range (2 mg/kg Q3W–10 mg/kg Q3W) or exposure and OS in metastatic melanoma and NSCLC. A strongly negative clearance–survival relationship with pembrolizumab in metastatic melanoma, not ameliorated by increased dose was, however, identified.^11^ The association of increased clearance with poorer survival outcomes has been replicated with nivolumab in advanced melanoma.^39^ Decreased OS in patients with higher clearance paralleled and, accordingly, was thought to be confounded by disease severity markers associated with end‐stage cancer‐cachexia syndrome, weight loss, and fall in albumin. The authors hypothesized that patients with rapid ICB clearance, increased cachexia, and poorer performance status were simply a reflection of a resistant disease cohort, rather than intrinsically causal of ICB failure.^11^, ^40^ Though biologically conceivable, this cachexia hypothesis relies on clearance calculated using a time‐dependent population PK model^38^ that included change in weight and albumin as some of a limited number of covariates to explain inter‐individual variability in PKs. Consequently, it is unsurprising that changes in albumin and weight have statistically significant effects on relationships between survival and clearance given that they were utilized to improve clearance estimation in the initial model. Given that changes in weight and albumin are time‐varying factors, captured up to week 9, they may be simply capturing deteriorating patients during treatment, explaining association with poorer OS. No other time variable covariates were included in this time‐dependent population PK model. Other groups^41^ have noted that discrete thresholds regarding baseline sum of the longest diameter of target lesions (BSLD), a proxy for tumor burden, were not mentioned in either clearance^11^ or population PK model.^38^ Given that clearance and clinical status at baseline and throughout treatment is associated with confounding BSLD, sufficient drug concentrations may not be reached at higher tumor burdens due to an intra‐tumoral sink effect on PK with increasingly advanced disease.^41^ Some patients, as identified by those with <median drug exposure and dramatically inferior OS in our work, may not reach effective exposure of pembrolizumab, leading to inferior outcomes.

Moving to potential future biomarkers, increased frequency of expression of TIM‐3 and CXCR6^+^ on CD4^+^ T cells in metastatic melanoma patients with PD was confirmed (Figure 5A). This correlates leukocyte trafficking with T cell dysfunction translationally and attaches clinical relevance via BOR. Co‐expression of CXCR6^+^ and HLA‐DR (*p* = .001) was upregulated in patients with PD (Figure 5) over those that had an objective response. This suggests that the co‐expression of CXCR6 and some T‐cell exhaustion markers on circulating T lymphocytes are potential biomarkers of disease progression. In this study, we could not determine where the exhausted T cells were being trafficked, only that they are present in the sample of PBMCs. Future studies focused on tumor tissue and PBMCs exhaustion markers before commencement of pembrolizumab and during the course of treatment may elucidate specific thresholds for chemokines and T‐cell exhaustion markers to identify those unlikely to benefit from single agent pembrolizumab.

The cohort size (*n* = 28) limits some of the statistical inferences of our findings. Non‐parametric correlation analysis found independent prognostic factor LDH at baseline (−.398, *p* = .004) was related to OS, which confirms previous studies. No other risk factors, apart from pembrolizumab exposure as discussed, were found to be associated with PFS or OS. We did not assess confounding factors associated with treatment outcomes and drug exposure such as BSLD and cancer cachexia surrogates (changes in albumin and body weight). Trough samples were taken at a variety of timepoints, preventing identification of a clear temporal relationship between variations in exposure and PD.

Variable time‐dependent clearance^38^, ^42^ of ICB and provocative signals of low drug exposure and inferior PFS and OS alongside similar work with other ICBs in NSCLC^14^ lead us to postulate that lower drug exposure may contribute to cause rather than effect of inferior outcomes. Clinically impactful inter‐patient PK variability is seen in a real‐world setting, and important limitations to the presumed effect of baseline advanced disease state and cachexia have been outlined. Modern frameworks for assessing the exposure–efficacy relationship within ICB monoclonal antibodies have moved from a simplistic one‐way correlation between independent and dependent variables, respectively, to a complex multi‐faceted set of interactions that likely are influenced multi‐directionally and by baseline prognostic characteristics.^43^

Given the large inter‐individual exposure variability, mooted exposure–survival relationship and availability of a reproducible assay, further prospective therapeutic drug monitoring studies are planned in order to establish whether low pembrolizumab drug exposure is a modifiable variable that may lead to improvement in OS. Upregulation of T‐cell exhaustion and leukocyte trafficking markers on CD4^+^ cells was associated with PD. This is the first work outlining these relationships with pembrolizumab.

## DISCLOSURE

AvdW and NAB receive funding for investigator‐initiated clinical trials from Merck KGaA & Bristol Myers Squibb that is not related to this study. All other authors have no relevant conflicts of interest.

## AUTHOR CONTRIBUTION

VN collected and interpreted data and wrote the manuscript; MCG conceived the project, collected and analyzed data and contributed to the manuscript, GCM collected clinical data, HM collected clinical data and reviewing imaging findings, AvdW collected and interpreted data and contributed to the manuscript, NAB supervised the collection of data, analyzed and interpreted data and wrote the manuscript.

## ETHICS APPROVAL AND CONSENT TO PARTICIPATE

This study was carried out in accordance with the recommendations of the National statement on ethical conduct in human research, National Health and Medical Research Council, Australia with written informed consent from all subjects. All subjects gave written informed consent, including consent for publication, in accordance with the Declaration of Helsinki. The protocol was approved by the Hunter New England Human Research Ethics Committee H‐2018–0159.

## Data Availability

The data that support the findings of this study are available from the corresponding author upon reasonable request.
